# Microbiome Sex-Related Diversity in Non-Muscle-Invasive Urothelial Bladder Cancer

**DOI:** 10.3390/cimb46040225

**Published:** 2024-04-19

**Authors:** Konrad Bilski, Natalia Żeber-Lubecka, Maria Kulecka, Michalina Dąbrowska, Aneta Bałabas, Jerzy Ostrowski, Aleksandra Dobruch, Jakub Dobruch

**Affiliations:** 1 Department of Urology, Centre of Postgraduate Medical Education, Independent Public Hospital of Prof. W. Orlowski, 00-416 Warsaw, Poland; jdobruch@cmkp.edu.pl; 2 Department of Gastroenterology, Hepatology and Clinical Oncology, Centre for Postgraduate Medical Education, 02-781 Warsaw, Poland; natalia.zeber-lubecka@cmkp.edu.pl (N.Ż.-L.); mkulecka@cmkp.edu.pl (M.K.); jostrow@warman.com.pl (J.O.); 3 Department of Genetics, Maria Sklodowska-Curie National Research Institute of Oncology, 02-781 Warsaw, Poland; michalina.dabrowska@nio.gov.pl (M.D.); aneta.balabas@nio.gov.pl (A.B.); 4 Sapienza University of Rome, 00185 Rome, Italy; oladobruch625@gmail.com

**Keywords:** microbiome, urothelial bladder cancer, sex-related diversity

## Abstract

Sex-specific discrepancies in bladder cancer (BCa) are reported, and new studies imply that microbiome may partially explain the diversity. We aim to provide characterization of the bladder microbiome in both sexes diagnosed with non-muscle-invasive BCa with specific insight into cancer grade. In our study, 16S rRNA next-generation sequencing was performed on midstream urine, bladder tumor sample, and healthy-appearing bladder mucosa. Bacterial DNA was isolated using QIAamp Viral RNA Mini Kit. Metagenomic analysis was performed using hypervariable fragments of the 16S rRNA gene on Ion Torrent Personal Genome Machine platform. Of 41 sample triplets, 2153 taxa were discovered: 1739 in tumor samples, 1801 in healthy-appearing bladder mucosa and 1370 in midstream urine. Women were found to have smaller taxa richness in Chao1 index than men (*p* = 0.03). In comparison to low-grade tumors, patients with high-grade lesions had lower bacterial diversity and richness in urine. Significant differences between sexes in relative abundance of communities at family level were only observed in high-grade tumors.

## 1. Introduction

Bladder cancer (BCa) remains the most common malignancy of the urinary tract [1]. It is three–four times more common in men than in women, but females present with a more advanced disease and have a less favorable prognosis [2,3]. A number of investigators tried to explain the sex gap and introduced several hypotheses including delayed diagnosis, diverse management, and different responses to cancer therapy [3,4,5]. Hematuria is the dominant sign of BCa, particularly when blood clots are found without concomitant lower urinary tract complaints. Initial diagnosis is based on imaging but the final diagnosis is confirmed after cystoscopy followed by transurethral resection of the bladder tumor (TURBT) with subsequent histopathological examination of the specimens [6,7]. Indeed, there are data reporting no differences in clinical symptoms between sexes, while the primary diagnostic approach differs, as shown in numerous studies [2]. It has been observed that a sex gap in the evaluation of hematuria exists [8]. However, the exact role of a particular feature among a variety of intermingled factors is yet to be fully understood. About 75% of cases of bladder cancer (BCa) are limited to the mucosa at the time of diagnosis, which is referred to as non-muscle-invasive bladder cancer (NMIBC), while the remaining cases have invaded deeper layers of the bladder wall or have already formed metastases (MIBC—muscle invasive bladder cancer) [9]. The latter indicates a poor prognosis, while the former is highly variable and can be classified into three main prognostic subgroups. The mainstay of the division is the stage of the bladder lesion followed by its grade [10]. In the majority of cases, low-grade disease has indolent clinical history, whereas high-grade cancers pose significant risk to patients. In patients with non-muscle-invasive bladder cancer grading continues to play a crucial role in treatment decisions. Multiple studies have demonstrated that low- and high-grade BCa exhibit distinct molecular characteristics and are closely linked to disease recurrence and progression [11]. Patients with high-grade Ta tumors have a similar lifelong risk of disease progression and death as those with T1 tumors [12]. Furthermore, Bree KK et al. suggest that regardless of the stage, all high-grade BCa should be regarded as the high-risk group of NMIBC, based on the analysis of the risk of disease progression following BCG immunotherapy [13]. The tumor differentiation grade has been shown by several studies to be a stage-independent prognostic factor in NMIBC [14]. Therefore, with respect to sex, we decided to differentiate our NMIBC cohort into high- versus low-grade bladder tumors.

Throughout recent years, extensive research has been devoted to genetic and molecular alterations in bladder cancer. Recent investigations have revealed distinct genetic patterns of BCa with correspondence to conventional pathologic grade and stage subgroups [15,16,17,18]. The studies involved both patients with MIBC and those with NMIBC. Briefly, most studies of low-grade papillary urothelial cancers show a few molecular events apart from deletions involving chromosome 9 and mutations of FGFR3 (fibroblast growth factor receptor 3) and HRAS (harvey rat sarcoma viral oncogene) [19,20]. In MIBC, many genetic abnormalities have been reported which involve dysregulations of several oncogenes and tumor suppressor genes like recently shown aberrations in p53/MDM2 (murine double minute 2), RB1 (retinoblastoma 1) and E2F3 (E2F transcription factor 3) [21]. The interest in BCa biology has burst since publication of the landmark study of the Cancer Genome Project (TCGA) [22]. It has been shown that bladder cancer is a molecularly heterogeneous disease, and a multitude of genomic changes were found. Recently, Liu et al. accomplished genetic profiling of high- as well as low-grade NMIBC [23]. Extensive genomic heterogeneity was noted among high-grade lesions. Patschan et al. have further divided 167 T1 high-grade NMIBC into three prognostic groups—urobasal, genomically unstable, and squamous-cell-carcinoma-like, with the last two related with significantly worse clinical outcomes [24]. Likewise, comprehensive transcriptomic assay of 460 NMIBC samples revealed again three different subgroups of NMIBC similar to the previously described urobasal, genomically unstable, and squamous-cell-carcinoma-like subgroups. High-risk lesions identified using these molecularly defined classifications are consistent with the high-risk tumors identified by the clinical European Organization for Research and Treatment of Cancer (EORTC) risk scores. According to genetic profiling of NMIBC lesions, ranging from carcinoma in situ to Ta and T1 stages, TERT promoter mutations (73%) and chromatin remodeling gene mutations (69%) were found to be the most common in the sequenced BCa samples irrespective of disease stage and grade implying a role of these in the early pathogenesis of BCa [25]. Mutations of FGFR3 or ERBB2 were found in 57% of lesions and were mutually exclusive. FGFR3 mutations were more often present in the low-grade BCa with frequency of 83%; ERBB2 mutations were only found in high-grade BCa. At the same time, the receptor tyrosine kinase/phosphatidylinositol 3-kinase pathway mutations were found in 79% of NMIBC tumors, with PIK3CA alterations prevalent in 26% of the lesions, 47% of the NMIBC lesions had mutations involving TP53 or the cell cycle pathway—these mutations were correlated with higher grades and stages of BCa [26]. Similarly, DNA damage repair (DDR) genes mutations were more often present in high-grade NMIBC tumors (30%) than among low-grade lesions (4%) [27]. The tumor mutational burden (TMB) correlated with the presence of DDR defect and was higher in the high-grade tumors with DDR gene mutations. ERCC2 alterations (17%) were the most commonly present DDR gene alterations [28]. The similar spread of DDR gene mutations and the degree of TMB in the high-grade NMIBC group to MIBC signifies a close genetic resemblance of high-grade NMIBC to MIBC. It is worth emphasizing that authors of the study reported cell cycle and damage response genes alterations; in particular, E2F1/2 and a wide range of E2F are mutated in high-grade NMIBC lesions [29]. Importantly, genes involved in embryonic development and morphogenesis (Hox genes, sonic hedge- hog [SHH], wingless-type MMTV integration site family, member 6 [WNT6], and wingless-type MMTV integration site family, member 10A [WNT10A]) were down-regulated in high-grade NMIBC tumors [30].

Recent advancements in molecular engineering have allowed researchers to explore the intricate connections between the host and the microbiome, which has been found to exist within a variety of its vital systems [31]. Not surprisingly, the role of the gut, pancreatic, and breast microbiome in carcinogenesis was later explored and multifactorial relationships were revealed [32,33]. As such, the urinary microbiome (UMB) has also been investigated and shown to play a significant role in BCa [34,35,36,37,38]. Susceptibility of females to urinary tract infections has set the ground for research on UMB in BCa sex diversities. Preliminary results suggest that as much as 30% of cancers would be related to microbial infection [39]. However, only few studies with a limited number of patients with BCa with respect to UMB were published. In addition, concurrent clinical data essential to draw any meaningful conclusion are scarce. Hence, we have designed research which aims at elucidating UMB metrics in both sexes diagnosed with NMIBC with specific insight into the cancer grade. The objective of this study was to compare sex-related diversities in microbiome signatures, resulting from 16S rRNA next-generation sequencing-based metagenomics analyses of midstream urine and paired bladder and tumor tissues among a cohort of individuals with histopathologically proven urothelial bladder cancer.

## 2. Materials and Methods

### 2.1. Patients

Informed written consent was obtained from individuals undergoing transurethral resection of the bladder tumor (TURBT) in our tertiary institution. Inclusion criteria were (1) >18 years of age; (2) no bladder catheter in place; (3) no symptoms or signs of an active urinary tract infection. Exclusion criteria included (1) inability to apply inclusion criteria, (2) refusal to give informed consent, and (3) muscle-invasive bladder cancer. The consent involved analysis of three different types of biological material: mid-stream urine, tumor sample, and healthy-appearing bladder mucosa.

Mid-stream urine collection was employed for both men and women. Patients were requested to collect a urine sample the day of TURBT. Participants were instructed on the proper midstream urine collection techniques to minimize external contamination. Morning urine samples were collected in sterile containers from the 41 studied patients (21 male, median age 82 and 20 female, median age 79). None of the patients presented clinical symptoms of urinary tract infection at the time of urine sampling or received antibiotics during the 72 days before urine collection. Samples were stored within 90 min at −80 °C until analysis. Similarly, small biopsy specimens were obtained from the cancerous samples of the urinary bladder and healthy-appearing bladder mucosa, collected at the time of TURBT. Tissue samples were obtained via transurethral resection of the bladder tumor procedure under sterile conditions. After collection in sterile Eppendorf tubes, tissues were frozen and stored at −80 °C until use.

A separate analysis concerned standard histologic subgroups of BCa including grade (low-grade and high-grade) and stage (Ta and T1) of the disease to avoid heterogeneity between known distinct BCa entities

### 2.2. Bacterial DNA Isolation

Urine genomic bacterial DNA isolation was performed using the QIAamp Viral RNA Mini Kit (QIAGEN, Hilden, Germany) according to the instruction for purification of bacterial DNA from urine and author modifications. Urine samples of 5 mL volume were centrifuged to concentrate; next, 140 µL of urine was taken and mixed with 540 µL of AVL buffer (QIAGEN, Hilden, Germany) to inactivate the numerous unidentified PCR inhibitors found in urine. After 10 min of incubation, 100% ethanol was added, and the mixture was transferred onto QIAamp Mini columns (QIAGEN, Hilden, Germany) and centrifuged. After purification using AW1 and AW2 buffers (QIAGEN, Hilden, Germany), DNA was eluted from the columns with nuclease free water. DNA samples were divided into 1.5 mL Eppendorf test tubes.

Cancerous samples and healthy bladder mucosa genomic DNA were extracted and purified using QIAamp DNA Mini Kits (QIAGEN, Hilden, Germany), according to the manufacturer’s instructions, as described previously [40]. The amount of extracted DNA from urine and bladder tissues were measured using Nanodrop ND-1000 spectrophotometer (Thermo Fisher Scientific, Waltham, MA, USA). Bacterial genomic DNA was stored at −20 °C until further analysis.

### 2.3. 16S rRNA Gene Analysis

Bacterial 16S rRNA libraries were constructed utilizing the Ion 16S Metagenomics Kit (Thermo Fisher Scientific, Waltham, MA, USA), which facilitates a comprehensive analysis of six distinct regions (V2, V3, V4, V6–7, V8, and V9). Additionally, an Ion Plus Fragment Library Kit (Thermo Fisher Scientific, Waltham, MA, USA) was employed for library preparation, following established protocols [41,42]. Hypervariable fragments of the 16S rRNA gene analysis of the urine, cancerous tissues, and healthy bladder mucosa microflora were performed using the Ion Torrent Personal Genome Machine (PGM) platform (Thermo FisherScientific, Waltham, MA, USA), as described previously [43]. Template preparation and sequencing of the barcoded libraries were performed using the Ion PGM™ Hi-Q™ View OT2 400 Kit and Ion PGM Sequencing 400 Kit (Thermo Fisher Scientific, Waltham, MA, USA) according to the manufacturer’s instructions.

### 2.4. Statistical and Bioinformatic Analysis

Unmapped BAM files were converted to FASTQ using Picard’s SamToFastq. Additional steps of the analysis were performed using Mothur software version 1.38 [44]. FASTQ files were converted to the FASTA format. Only sequences that were 200–300 bases in length, with an average base quality of 20 in a sliding window of 50 bases, and a maximum homopolymer length of 10, were included. Chimeric sequences were identified using the UCHIME [45] algorithm using default parameters, with internal sequence collection as the reference database. Chimeric sequences were removed and the remaining 16S rRNA sequences were classified using the Wang method and the SILVA [46] bacterial 16S rRNA database for reference (release 138); the bootstrap cut-off was 80%. Counts on the genus level were obtained with MEGAN5. FDR-adjusted [47] *p*-values ≤ 0.05 were considered statistically significant. Alpha diversity analysis was performed using Shannon and Chao1 indexes as indicators. PCoA was performed using the Bray–Curtis index as a distance measure. ANOSIM was used to test the significance of clustering patterns. The differential abundance of taxa was assessed using the metagenomeSeq method for taxa with at least 10× sequencing depth.

## 3. Results

Bacterial DNA was extracted from a total of 112 bladder cancer patients. On average, there were 229 thousand reads classified per sample (median: 185 thousand). An average of 12% (median—3%) of reads were not classified as bacteria—such reads were removed from further analysis. Only samples that yielded sufficient DNA and met the sequencing quality criteria were ultimately included in the final analysis. As a result, the final cohort included 41 paired triplets of midstream urine, tumor samples, and healthy-appearing mucosa obtained from urothelial BCa patients. Clinical and histopathological characteristics of the study cohort are summarized in Table 1.

There were 2153 taxa discovered among all samples: 1739 in tumor tissue, 1801 in bladder tissue, and 1370 in urine. Counts on the genus level were obtained with MEGAN5. Proteobacteria phylum was the dominant phylum in the largest number of samples in every tissue tested (63% for normal tissue, 48% for tumor tissue, and 46% for urine). The Proteobacteria had the highest mean abundance in every group (around 38%). Other abundant phyla were *Firmicutes*, *Bacteroidota*, and *Actinobacteriota* (Supplementary Figure S1). On a family level, *Streptococcaceae* were the dominant family in 45% and 39% tumor and normal tissue samples, while *Enterobacteriaceae* were dominant in 32% urine samples.

**Figure 1:**
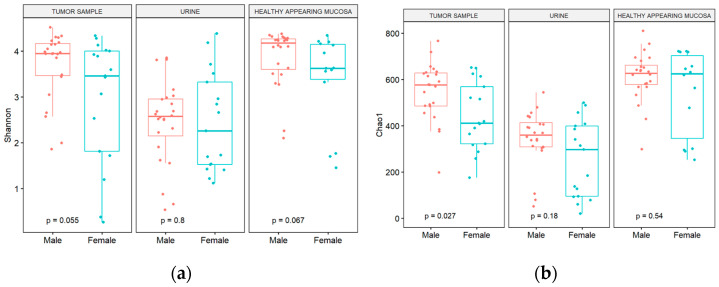
(**a**) Shannon diversity; (**b**) Chao1 index at genus level from urine, mucosa, and tumor samples obtained from patients stratified by sex.

Figure 1a shows the observed Shannon diversity at the genus level, comparing the sex and sample type groups. Regarding sex, there were no statistically significant differences between male and female patients in the Shannon diversity index; although in tumor and bladder mucosa samples, the adjusted *p*-value nearly reached the level of statistical significance (*p* = 0.055 and *p* = 0.067, respectively) with female patients presenting with a less diverse microbiome. Contrary, Chao1 index values differentiated male and female tumor samples, with women having a smaller taxa richness (Figure 1b).

**Figure 2:**
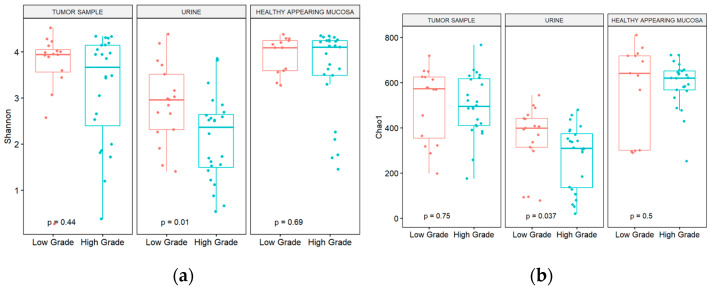
(**a**) Shannon diversity; (**b**) Chao1 index at genus level from urine, mucosa, and tumor samples obtained from patients stratified by tumor grade.

As far as tumor grades are concerned, patients with high-grade tumors presented with lower bacterial diversity and richness in urine (Figure 2a,b). Considering both sex and grade, women with high-grade tumors presented with significantly lower diversity and richness in cancer samples than men (adjusted *p* value = 0.019 and 0.0087 for Shannon diversity and Chao1 index, respectively). In female urine, there was also a statistically significant difference in diversity between high- and low-grade tumors (adjusted *p* value = 0.025 Shannon diversity and Chao1 index, respectively), while in male urine there was no difference in richness between different tumor grades.

**Figure 3:**
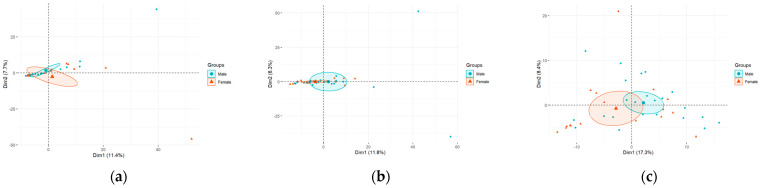
Principal component analysis (PCA) of bacteria according to sex at the genus level in urine (**a**), bladder mucosa (**b**), and tumor sample (**c**).

There were statistically significant differences between sexes in community distances as measured using Bray–Curtis index. These variations applied to every sample tested (Figure 3a–c). Moreover, no statistically significant differences were observed between different tumor grades.

Using metagenomeSeq, we were able to find differentially abundant specific OTUs in both sexes with respect to the disease grade. In comparison with sexes, large differences were seen. There were 92, 120, and 98 differential taxa in bladder mucosa, tumor samples, and urine, respectively (Supplementary Table S1). In bladder mucosa and tumor samples, 81 and 107 taxa were underrepresented in women, which reflects lower bacterial diversity observed in female patients’ tissues (Supplementary Table S1). While, in male urine samples, 45 taxa were overrepresented, including abundant genera *Campylobacter*, *Sphingobium*, and *Haemophilus* (Supplementary Table S1). In high-grade tumors, sex-related differences were less pronounced in bladder mucosa and urine samples, with only 20 and 9 differential taxa, respectively. While, in the tumor tissues, as many as 153 differentiating bacteria have been identified (Supplementary Table S2). Bacteria overrepresented in high-grade female tissues included *Salmonella* genus (both in bladder mucosa and tumor samples), *Romboutsia* (bladder mucosa only), and *Enterobacter* (tumor sample only) (Supplementary Table S2). What is more, in cancerous tissue, 149 taxa were overrepresented in men with high-grade BCa, including abundant genera *Aeribacillus*, *Peptococcus*, *Alcaligenes*, *Actinomyces*, *Pseudomonas*, *Acinetobacter*, *Proteus* (Supplementary Table S2). Interestingly, no statistically significant differences were present in low-grade tumors in tumor and urine samples, with only minimal differences in bladder mucosa (*Rhodocyclaceae C39* and *Cellvibrionales unclassified*) (Supplementary Table S3).

## 4. Discussion

To the best of our knowledge, the study provides the first sex-based profile of the urinary microbiome in patients with urothelial BCa with respect to the disease grade. Both alpha and beta diversity showed differences between males and females and between high- and low-grade diseases as well. It further highlighted the polymicrobial composition of human urine, with marked individual variations. Chao1 and Shannon indexes of urine samples were lower in high-grade tumor patients suggesting diminished abundance and diversity of microflora in this subgroup of BCa patients. Chao1 index values of tumor samples show smaller taxa richness in women than in men, but at the same time healthy-appearing bladder mucosa presents no sex-differences in this regard. Finally, women diagnosed with high-grade tumors are found to have significantly lower diversity and richness of their urinary microbiome than their male counterparts.

Persistent inflammation reduces the presence of beneficial microbial community members involved in maintaining epithelial health and immune homeostasis and increases some opportunistic pathogens intensifying chronic inflammation [48]. Inflammation may also have an impact on carcinogenesis by altering functions of the mucosal barrier and translocation of bacteria to tumor tissues [49]. Cancer is a disease of genome, and gradually accumulated mutations in the oncogenic gene shape the growth rate of colonic epithelial cells, reduce their susceptibility to cell death, endow them with metabolic specializations, and confer on them abilities to commandeer immune cells to further promote growth and spread [50]. Bacteria can directly damage the host DNA via specific substances called “genotoxins”, such as colibactin produced by some E. coli strains or indirectly by generating reactive oxidative species [51]. Other pathogenic microorganisms influence host signaling pathways, for example, the Wnt/β-catenin pathway which is altered to support cell proliferation in many types of cancers [52]. Extracellular superoxide from Enterococcus faecalis can induce chromosomal instability in human cells [53]. *Escherichia coli* of the phylogenetic group B2 induces DNA double-strand breaks in intestinal epithelial cells [54]. Exposure of intestinal epithelial cell lines to Bacteroides fragilis toxin results in an increased cellular proliferation, which is mediated by the elevated expression of the c-Myc oncogene [55]. However, a majority of studies were focused on the relationship between gut microbiota and colorectal carcinogenesis where the gut microbiota potentially contributes to a cancer risk via three major routes: (1) altering host cell proliferation or turnover, (2) influencing immune function, and (3) metabolizing ingested and host-derived products [56,57,58,59].

The observed differences in taxa richness between men and women in our cohort, particularly highlighted by the Chao1 index indicating smaller taxa richness in women, prompt a deeper exploration of their implications and potential factors contributing to these sex-specific distinctions. Firstly, the Chao1 index, a measure of species richness, suggests that women harbour a less diverse microbial community in the bladder compared to men. This discrepancy in microbial richness could be indicative of differing microbial colonization patterns, immune responses, or hormonal influences between sexes [60,61,62]. One implication of the reduced taxa richness in women is the potential alteration of microbial community dynamics within the bladder microenvironment. A less diverse microbial ecosystem may lead to decreased microbial competition, altered metabolic pathways, and potentially compromised host–microbiome interactions, all of which could influence disease susceptibility and progression. Several factors may contribute to these sex-specific differences in the bladder microbiota. Hormonal fluctuations, particularly oestrogen and progesterone levels, have been shown to influence the composition and stability of the urinary microbiome [60]. Additionally, anatomical disparities between male and female urinary tracts, such as urethral length and proximity to the perineum, may also play a role in shaping microbial communities [63]. The relevance of these sex-specific differences to bladder cancer progression and treatment lies in their potential impact on disease development, response to therapy, and overall patient outcomes. Variations in microbial composition and diversity could affect tumour microenvironment characteristics, immune modulation, and therapeutic efficacy [64]. Understanding these differences may lead to personalized approaches for bladder cancer management, including tailored interventions targeting the microbiome to improve treatment outcomes. In conclusion, the observed differences in taxa richness between men and women, particularly highlighted by the Chao1 index, underscore the importance of considering sex-specific factors in understanding bladder microbiome dynamics and their implications for cancer progression and treatment. Further research elucidating the underlying mechanisms driving these differences is crucial for understanding bladder cancer pathogenesis and developing targeted therapeutic strategies.

Decreased diversity of specific microbiomes has been correlated to a number of diseases, including colorectal and cervical cancers, skin, pulmonary tract and lower urinary system dysfunctions as well [65,66,67,68,69,70]. The interplay among composite communities of bacteria and the host is thought to influence its immunity by modulating multiple immunologic pathways. In our study, women with high-grade tumors presented with significantly lower diversity and richness of their microbiome within cancer samples compared to their male counterparts. Furthermore, the microbiome in midstream urine in females with high-grade disease was less diverse than in females with low-grade tumors. Several investigations have shown reduced diversity and richness of the urine microbiota in patients with bladder cancer when compared to patients with benign urological conditions. Hrbáček J et al. revealed differences between BCa patients and those with benign urological conditions exploring catheterized urine [71]. Pederzoli F. et al. found no differences in microbiome composition in terms of overall diversity or composition in urine specimens collected from 12 male patients diagnosed with bladder cancer and from 11 healthy individuals [37]. The latter results are obviously hampered by the limited number of cases. In patients with colorectal cancer, several studies revealed lower microbial diversity in cancerous tissues compared to normal-appearing mucosa [72,73]. Furthermore, it has been demonstrated that lower diversity of gut microbiota is associated with a poorer response to chemoradiation [69]. In cervical cancer patients, significant differences in α and β diversity between patients and healthy controls were also observed [74]. Therefore, we speculate that dysbiosis of the microbiota might be implicated in carcinogenesis, therapy-related side effects and treatment outcomes in bladder cancer, and may (at least partially) explain sex-specific differences in tumor biology.

The findings regarding bacterial diversity and richness in urine samples in our cohort especially concerning tumor grade, offer significant insights into the diagnosis and prognosis of non-muscle-invasive bladder cancer (NMIBC). Variations in bacterial diversity and richness across tumor grades could reflect differences in tumor microenvironment characteristics and immune responses. Differences in microbial composition between tumor grades may provide additional information for stratifying patients based on disease severity and guiding treatment decisions. Incorporating urinary microbiome analysis into diagnostic protocols may enhance the accuracy of NMIBC detection and improve early intervention strategies. Targeting specific microbial signatures associated with tumor grading could offer novel therapeutic avenues for modulating the tumor microenvironment and improving treatment outcomes. Further validation and standardization of urinary microbiome-based diagnostic and prognostic assays are warranted to ensure their clinical utility and reliability.

We noted differences in the relative abundance of specific taxa between sexes with respect to the grade. It is noted that certain bacterial taxa may be associated with different tumor grades, suggesting a potential link between microbial composition and bladder cancer progression. In women with high-grade cancers, *Salmonella* and *Enterobacter* were overrepresented in a cancerous specimen when compared to males. These microorganisms were suggested to be involved in carcinogenesis across several malignancies. *Salmonella typhi* has been shown to be associated with hepatobiliary and colon cancers, through the activation of the Wnt/β-catenin pathway [75]. Mansour et al. demonstrated that bladder tissue samples exhibited a higher prevalence of *Akkermansia*, *Bacteroides*, *Clostridium sensu stricto*, *Enterobacter*, and *Klebsiella* compared to urine samples [76]. Additionally, in the study conducted by Pederzoli et al., the *Enterobacteriaceae* family was found to be more prevalent in females [37]. Notably, bacteria from the *Enterobacteriaceae* family are known to produce toxins such as colibactin, which directly contribute to bladder tumorigenesis.

In our study, in male urine samples, 45 taxa showed higher representation, including the prevalent genera *Campylobacter*, *Sphingobium*, and *Haemophilus*. Bučević Popović V. et al. revealed similar findings [77]. Relative enrichment of Campylobacter has been observed in urine in BCa patients when compared to healthy controls. *Haemophilus* has been overrepresented in patients with muscle-invasive disease when compared to non-muscle-invasive bladder cancer in a study by Bi H. et al. [38]. Conversely, *Sphingobium* may play a protective role. Due to its extra ability to metabolize estrogen, it may have favorable influence on outcomes in breast cancer, especially estrogen-positive breast cancer [78,79]. However, the exact roles the pathogens may play in bladder cancer pathology remain to be seen.

In comparison with the female cohort, men with high-grade BCa were found to have at least 149 bacterial taxa that were more abundant in the cancerous specimen. These include *Aeribacillus*, *Peptococcus*, *Alcaligenes*, *Actinomyces*, *Pseudomonas*, *Acinetobacter*, *Proteus*. Xu et al. noted that certain genera, including *Streptococcus*, *Pseudomonas*, and *Anaerococcus*, were more commonly observed in patients diagnosed with urothelial carcinoma [80]. Reports indicate that bladder cancer patients exhibited increased levels of *Actinomyces*, whereas the control group showed enrichment of *Bifidobacterium*, *Streptococcus*, and *Lactobacillus* [43]. Liu et al. uncovered elevated relative abundances of various microbial genera, including *Acinetobacter* and *Anoxybacillus*, in cancerous tissues compared to normal tissues [35]. Wu et al. demonstrated higher bacterial richness and the enrichment of certain bacterial genera, including *Acinetobacter*, *Anaerococcus*, and *Sphingobacterium*, alongside a decrease in others, including *Serratia*, *Proteus*, and *Roseomonas*, within the bladder cancer group compared to the non-cancer group [81]. By understanding how microbial communities vary between sexes and their impact on tumor characteristics, we can potentially tailor treatments to individual patients. This personalized approach could lead to more effective and targeted therapies, ultimately improving outcomes for bladder cancer patients.

It is of note that significant differences in the microbiome diversity of female and male high-grade BCa were observed in cancerous samples only. Midstream urine and healthy tissue microbiome analysis did not confirm the findings. Our results suggest that urine samples may not mirror microbiota composition at the site of the cancer. Furthermore, we believe the tumor microenvironment influences microbiome arrangement and leads to the overrepresentation of a few specific microorganisms. Pederzoli F. et al. provided detailed characterization of the BCa urinary microbiome in urine and in bladder cancerous and non-cancerous tissue specimens [37]. They found different clustering of urine versus the tissue microbiome in men and women, supporting evidence for a different bacterial composition of these two environments. In parallel to BCa, in colorectal cancer, the distribution of the microbiome differs significantly between normal and tumor tissues [82]. The fecal microbiome is more variable and inconsistent probably due to numerous factors that may influence its composition including diet or lifestyle [83,84]. Hence, we believe one may find more reliable information on the microbiome through tissue-based sample exploration only, rather than upon more diverse microbiota of midstream urine. While the use of midstream urine samples for microbiome studies may have advantages due to an ease of collection and non-invasive nature, their composition may not accurately represent the more stable cancer tissue microbiota and the mucosal interactions across the bladder.

Bladder cancer progression involves a complex interplay of various molecular and cellular mechanisms [85]. One potential mechanism involves the direct interaction between specific microbial species and the bladder epithelium, which could lead to chronic inflammation and tissue damage, ultimately promoting tumor initiation and progression. Furthermore, the microbiome can modulate immune responses within the bladder microenvironment, influencing the balance between pro-inflammatory and anti-tumor immune pathways. Dysregulation of these immune responses by the microbiome may contribute to immune evasion by tumor cells and facilitate tumor growth and metastasis. Additionally, microbial metabolites and signaling molecules produced within the bladder may impact tumor cell behavior, including proliferation, invasion, and response to therapy. These metabolites can also influence immune cell function and the tumor microenvironment, further shaping the course of bladder cancer progression.

Clinical applications of research on microbiome in bladder cancer are very limited. In patients with recurrent bladder cancer relative abundance of *Lactobacillus* was lower than in those without disease failure, though the difference between the two groups did not reach the level of significance [86]. Seow SW et al. demonstrated the *Lactobacillus* species exerted anti-proliferative effects in the bladder cancer cell line [87]. Oral administration of *Lactobacillus*, in addition to epirubicin instillations, was demonstrated to decrease the risk of recurrence at three years when compared to intravesical therapy only in a randomized controlled trial by Naito S et al. [88]. In conclusion, oral intake of *Lactobacillus* may be helpful in preventing bladder cancer recurrence. Among those with BCa subjected to BCG therapy, overrepresentation of *Corynebacterium* and *Pseudomonas* was found in the responding subgroup of patients. The presence of Aerococcus species might indicate a less favorable response to BCG, whereas the presence of Ureaplasma and Escherichia/Shigella could suggest a more apositive response to BCG. Additional research is needed to clarify and verify the importance of alterations in the bladder microbiome. Specific microbial signatures can be used for various purposes, such as predicting the response to therapeutics [89].

Building on the emerging evidence linking the microbiome to cancer biology, future research could explore microbiome-targeted therapies for bladder cancer. Our study suggests that the bladder microbiome may serve as a potential source of biomarkers for bladder cancer diagnosis and prognosis. Future research could aim to validate and refine these findings in larger cohorts of NMIBC patients. Strategies such as probiotics, prebiotics, dietary interventions, and microbiota transplantation could be investigated for their potential to modulate the bladder microbiome and influence bladder cancer outcomes. Clinical trials evaluating the efficacy and safety of microbiome-based interventions in bladder cancer patients are warranted to translate these findings into clinical practice. Overall, future research efforts should aim to leverage the findings of our study to advance our understanding of the role of the microbiome in bladder cancer and translate these discoveries into clinically actionable strategies for improving patient outcomes.

We acknowledge several limitations of our study. Our study design is observational in nature, which precludes establishing causality between the urinary microbiome and bladder cancer outcomes. While we have identified associations between microbial profiles and disease parameters, further longitudinal or interventional studies are necessary to elucidate the causal relationships and underlying mechanisms. The precision of our estimates is still limited by a moderate sample size. In addition to the intrinsic limitations of the amplicon-based microbiome approach, we could not control for patient-specific factors, such as race, diet, or exposure to environmental carcinogens. We could not exclude the potential bias introduced by previous TURBT or by previous antibiotic therapies, although their impact on the bladder and urinary microbiome is currently unclear. Furthermore, we collected the samples only once and prior to surgery, so we cannot comment on the microbial stability over time. In our study, diversity of the microbiome in BCa patients differed between sexes, specifically in high-grade disease. Based on our research, we may assume that urinary microbiota consists of groups of taxa that are the core and are being rarely lost and additional groups of co-occurring microbiota that are associated positively or negatively with cancer. A lack of protective taxa can lead to the development of high-grade disease. Therefore, many types of bacteria are being studied in bladder cancer. Prospective studies are needed to disentangle the association between cancer development and microbial dysbiosis, as well as the possible role of these bacterial communities in the metabolism of carcinogenic compounds present in the urinary tract. We consider our data relevant for the development of future approaches for microbiota-based biomarkers of BCa and for microbiome modulation to boost therapy efficacy. Our study makes a preliminary exploration of the association of urinary microbiota and clinical features of bladder cancer and their association with sex. By uncovering differences in the bladder microbiome between male and female NMIBC patients, we have expanded our understanding of the interplay between microbial communities and cancer biology in this specific population. Sex-related differences in the bladder microbiome may reflect underlying biological mechanisms, such as hormonal influences, immune responses, and genetic factors, which contribute to bladder cancer development and progression. Accordingly, developing a predictive testing based on the urobiome composition could contribute to a personalized medicine approach in the future.

## 5. Conclusions

Bladder cancer persists as a malignancy affecting both men and women. Our study reveals the significant diversity of the bladder microbiome in both sexes diagnosed with non-muscle-invasive BCa, especially in those with high-grade cancers. The differences among patients with low- and high-grade tumors, as well as women and men may be used in the future studies to close the sex gap in bladder cancer.

## Figures and Tables

**Table 1:** Clinical features of the study cohort.

	Male	Female
Number	21 (51%)	20 (49%)
Mean age at TURBT	82	79
Low-grade	11 (52%)	12 (60%)
Non-invasive (Ta)	7 (33%)	8 (40%)
Invasive (T1)	4 (19%)	4 (20%)
High-grade	10 (48%)	8 (40%)
Non-invasive (Ta)	4 (19%)	3 (15%)
Invasive (T1)	6 (29%)	5 (25%)

## Data Availability

The raw data supporting the conclusions of this article will be made available by the authors on request.

## Supplementary Materials

The following supporting information can be downloaded at https://www.mdpi.com/article/10.3390/cimb46040225/s1. Supplementary Figure S1: Relative abundance of bacterial communities at phylum level in tumour sample, urine and healthy-appearing mucosa. Supplementary Table S1: Intergroup sex differences in the distribution of bacterial taxa across bladder mucosa, tumor tissues and urine samples. Supplementary Table S2: High-grade tumors sex-related differences in bladder mucosa, tumor tissues and urine samples. Supplementary Table S3: Low-grade tumors sex-related differences in bladder mucosa, tumor tissues and urine samples.
