# Supplementary figures and images for: Microbiome Sex-Related Diversity in Non-Muscle-Invasive Urothelial Bladder Cancer
Source: Curr Issues Mol Biol. 2024 Apr 19;46(4):3595–609. doi: 10.3390/cimb46040225 (PMC11048804; doi:10.3390/cimb46040225)

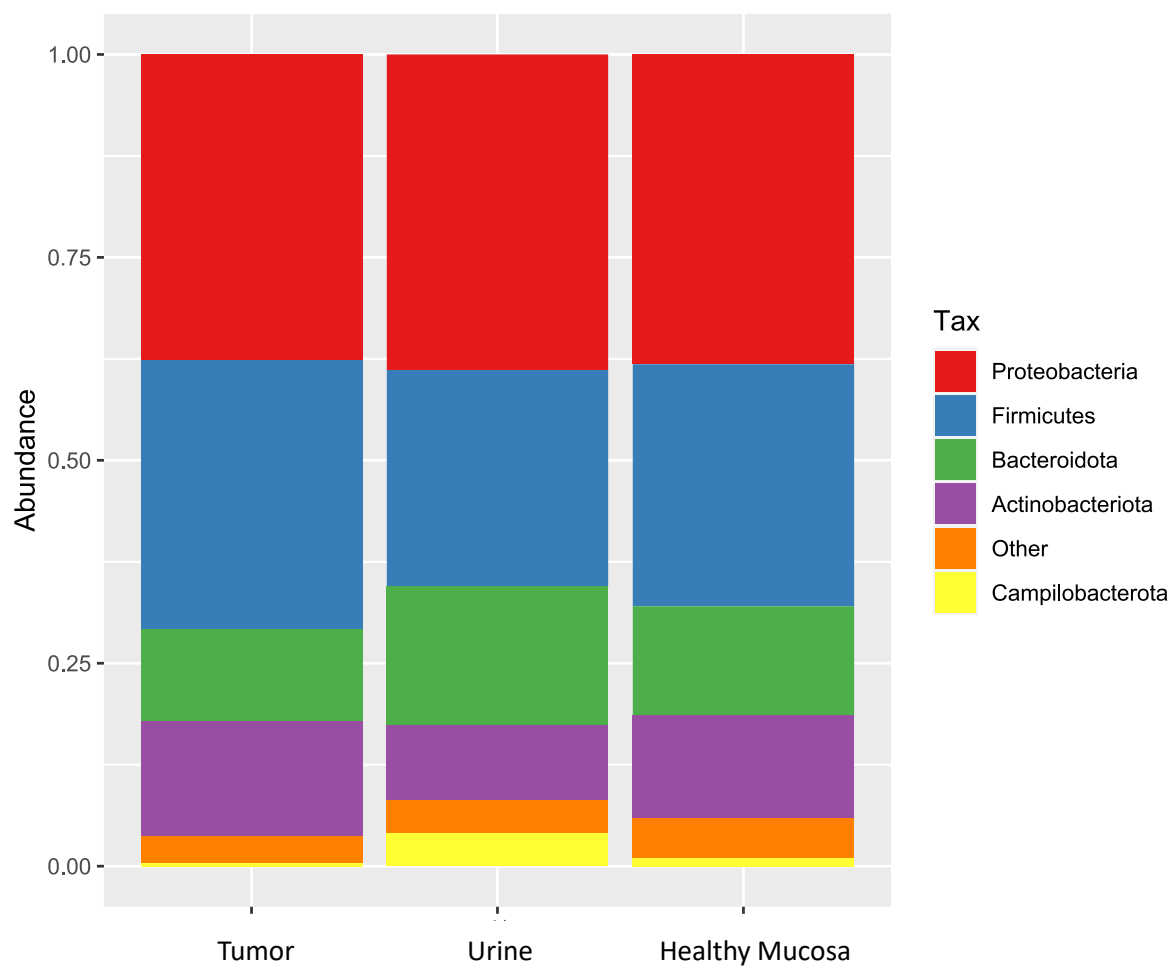

Supplement: Supplementary file 1 [file cimb-46-00225-s001.zip › Supplementary Figure S1.pdf]
